# Will More of the Same Achieve Malaria Elimination? Results from an Integrated Macroeconomic Epidemiological Demographic Model

**DOI:** 10.4269/ajtmh.19-0472

**Published:** 2020-09-21

**Authors:** Richard D. Smith, Marcus R. Keogh-Brown, R. Matthew Chico, Michael T. Bretscher, Chris Drakeley, Henning Tarp Jensen

**Affiliations:** 1 College of Medicine and Health, University of Exeter, Exeter, United Kingdom;; 2 Faculty of Public Health and Policy, London School of Hygiene and Tropical Medicine, London, United Kingdom;; 3 Faculty of Infectious and Tropical Diseases, London School of Hygiene and Tropical Medicine, London, United Kingdom;; 4 Department of Infectious Disease Epidemiology, MRC Centre for Outbreak Analysis and Modelling, Imperial College, London, United Kingdom;; 5 Department of Food and Resource Economics, Faculty of Science, University of Copenhagen, Frederiksberg, United Kingdom

## Abstract

Historic levels of funding have reduced the global burden of malaria in recent years. Questions remain, however, as to whether scaling up interventions, in parallel with economic growth, has made malaria elimination more likely today than previously. The consequences of “trying but failing” to eliminate malaria are also uncertain. Reduced malaria exposure decreases the acquisition of semi-immunity during childhood, a necessary phase of the immunological transition that occurs on the pathway to malaria elimination. During this transitional period, the risk of malaria resurgence increases as proportionately more individuals across all age-groups are less able to manage infections by immune response alone. We developed a robust model that integrates the effects of malaria transmission, demography, and macroeconomics in the context of *Plasmodium falciparum* malaria within a hyperendemic environment. We analyzed the potential for existing interventions, alongside economic development, to achieve malaria elimination. Simulation results indicate that a 2% increase in future economic growth will increase the US$5.1 billion cumulative economic burden of malaria in Ghana to US$7.2 billion, although increasing regional insecticide-treated net coverage rates by 25% will lower malaria reproduction numbers by just 9%, reduce population-wide morbidity by −0.1%, and reduce prevalence from 54% to 46% by 2034. As scaling up current malaria control tools, combined with economic growth, will be insufficient to interrupt malaria transmission in Ghana, high levels of malaria control should be maintained and investment in research and development should be increased to maintain the gains of the past decade and to minimize the risk of resurgence, as transmission drops.

## INTRODUCTION

Malaria was responsible for an estimated 435,000 deaths worldwide in 2017.^1^ This is a public health tragedy by any measure, but it does represent a decline of 50% since 2000.^1,2^ Progress has been attributed primarily to increasing the coverage of malaria control interventions. However, malaria is affected by many complex and interrelated factors and, although it is believed that urbanization and economic development may also have played a role,^2^ the size of this influence and their potential contribution toward the goal of elimination is unknown. In 2007, Bill and Melinda Gates, joined by the then director general of the WHO, Margaret Chan, declared that malaria eradication was possible and should be an international goal,^3^ one which has since been pursued by the malaria community.^4^ However, malaria eradication is not new terrain. Many previous efforts have been confounded and have led to calls for malaria elimination programs to establish links across research disciplines beyond epidemiology to include expertise on economic activities and population movements.^5^

The WHO’s Global Malaria Eradication Programme operated from 1955 to 1969 and relied heavily on dichlorodiphenyltrichloroethane to interrupt transmission, ultimately failing to rid malaria from high-transmission settings as partial success was commonly followed by resurgence. Cohen et al.^6^ identified 75 instances of failed attempts to eliminate malaria in 61 countries since the 1930s, attributable to multiple factors including the diversion of domestic and external resources away from malaria elimination efforts, as well as 19 cases in which changes in land use for commercial purposes contributed to resurgence. When the Global Malaria Eradication Programme was officially suspended, national malaria programs in Africa and Asia were largely left with compromised capacity for malaria control because of years of singular focus on malaria eradication. Then, during the global economic crises of the early 1970s, malaria-endemic countries adopted policies to stimulate economic growth that included aggressive encroachment into forested lands for commercial enterprise. These economic incentives resulted in population movement, increasing exposure to vectors carrying malaria and triggering an epidemic resurgence. As a result, private demand for preventive and curative use of chloroquine increased, driving drug pressure on natural selection that accelerated chloroquine resistance. This completed a cascade of adverse consequences that resulted from having neglected malaria control strategies after concerted elimination efforts, a sequence of events that had a significant influence on public and private demands for malaria interventions, population demographics, and the epidemiology of malaria.^5^ Present-day malaria control efforts may be reaching a critical juncture which is not so different from the past.^7^

Recent years have witnessed remarkable levels of funding for Africa from national and international sources to combat malaria; annual funding more than doubled from US$1.3 billion in 2005 to US$3.1 billion in 2017.^1^ However, it was estimated that global investment in malaria control and elimination by governments of malaria-endemic countries and international partners decreased to US$2.7 billion in 2018, well short of the US$5.0 billion required to meet the elimination targets set by the Global Technical Strategy for Malaria.^8^ In addition, recent reports indicate that progress toward elimination may have stalled and the burden of malaria in some countries may even be returning to 2010 levels.^7^ In light of this evidence, questions must be answered. Is the chance of elimination higher today than previous attempts? If so, what is different now? Are recent studies correct that using long-lasting insecticide-treated nets (ITNs) in combination with indoor residual spraying may not, by themselves, be sufficient to eliminate malaria?^9^ Or are there indirect factors which, if captured in parallel with malaria interventions, make elimination with existing tools more feasible? For example, questions remain concerning the role that broader influences of economic activities, population demographics, and private demand for interventions might play in current elimination strategies. Can malaria elimination be achieved directly by continuing to scale up the use of existing interventions, such as ITNs, to achieve greater population coverage, and indirectly by economic growth? Alternatively, if current efforts to reduce malaria transmission fall short of elimination, and if the recent reduction in research and development funding for malaria vaccines and drugs continues,^1^ might the decrease in acquisition of semi-immunity against malaria, at the population level, from years of intensified control, produce a tinderbox of risk that translates into a demographic shift of disease that will afflict all ages?

In the absence of a silver bullet to accomplish elimination, answering these questions is important and should be prioritized to guide the design of optimum control and elimination strategies. However, at this pivotal point for investment where funding for malaria interventions worldwide has plateaued,^8^ and when some model estimates from 2014 to 2016 show an increase in the number of malaria cases in more than 70% of the high-burden countries where per capita funding for the population at risk had previously reduced,^7^ finding answers to the important policy questions highlighted earlier is as urgent as it is complex. Assessing the potential of economic growth or scaling up the use and coverage of existing interventions to achieve malaria elimination first requires understanding the interaction and feedback loops^5^ between epidemiology, demography, and economics to enable development of models or tools that can quantify this potential. These models must capture multiple perspectives and simultaneously include spillover and feedback effects which may enhance or confound intervention efforts.

Many of the multiple factors which affect progress toward malaria elimination interact with each other, and some are exogenous to intervention strategies. For example, there is strong evidence that *Plasmodium falciparum* (*Pf*) malaria significantly impacts economic development and growth.^10,11^ However, economic development may also affect *R*_0_ (the expected number of secondary infections produced by each infected human in the absence of control or acquired immunity)^12,13^ as development-induced demographic changes may result in reduced human–vector contact.^14,15^ Therefore, capturing this bidirectional relationship in a model requires a specification of feedback between model components.

Research also indicates that economic growth could stimulate private demand for ITNs and be an external driver for reductions in the controlled reproductive number, *R*_C_ (which accounts for the presence of interventions).^16^ However, evidence to quantify the strength of interaction between growth and ITN uptake is lacking. The impact of economic development on malaria transmission is determined by “private” behavior, that is, the reaction of households to income growth, including internal migration (rural to urban) and the personal uptake of malaria interventions that are distinct from malaria control measures delivered through public channels. Capturing these connections is, therefore, important for policy analysis.

Studying the effect of these interactions is as complex as it is important, and the bar to this kind of cross-disciplinary policy analysis has previously been the technical difficulty of integrating models which simultaneously capture all of these elements together with their dynamic feedback effects. However, previous applications of computable general equilibrium (CGE) models in the context of HIV/AIDS,^17^ health co-benefits of green house gas reduction strategies,^18^ and dietary change^19–21^ have advanced the level of complexity and use of these models in a health context, and the tool we developed for this study demonstrates that full integration of models is possible. Furthermore, its utility is illustrated in analyses of various scenarios for future economic growth and scaling up of interventions. Details underlying our analytical tool, including the setup of this tool for the current application to *Pf* malaria data from Ghana, are publicly available elsewhere.^22^

We capture the potential public health consequences of failing to eliminate malaria after having intensified control efforts to historic levels, and by analyzing changes in future economic growth and scaling up of interventions, our study provides a means to measure the feasibility of the Ghana Health Service Resource Mobilization plan’s ultimate goal to achieve “self-sufficiency in funding of malaria elimination by 2030.”

**Figure 1.**
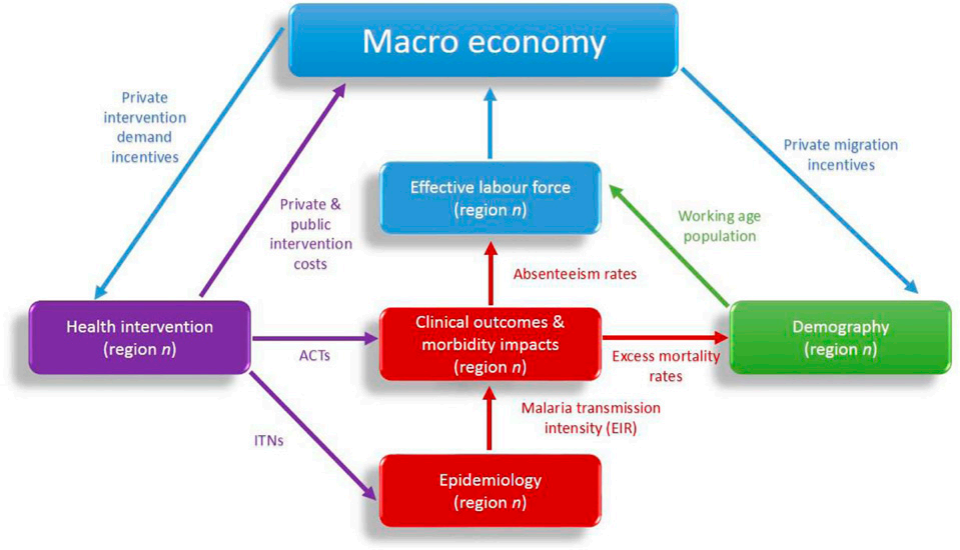
Diagram of epidemiological demographic macroeconomic malaria model framework and feedback effects between the macroeconomy and regional sub-models. This figure appears in color at www.ajtmh.org.

As illustrated in Figure 1, we use our integrated model, with its regional epidemiological models of malaria transmission and demographic models embedded as sub-models within a society-wide macroeconomic framework, to perform analyses of both endogenous private behavior and exogenous public interventions. We model the full circular impacts of income-driven population movement and malaria intervention adoption on regional economic and epidemiological outcomes including changes in *R*_C_. For the epidemiological component, we replicated the Swiss Tropical Institute (STI) malaria model for a typical sub-Saharan African nation, Ghana,^23^ with hyperendemic transmission. The results presented in the following use this model to determine the potential for existing interventions, alongside economic development, to achieve malaria elimination in Ghana.

## MATERIALS AND METHODS

### Macroeconomic model.

The policy tool at the heart of this analysis, which has been developed to perform integrated assessment, is a CGE model, specifically the International Food Policy Research Institute standard CGE model.^24^ The sectoral macroeconomic model captures the cost-minimizing and profit-maximizing behavior of producers, the consumption and saving behavior of households and government, taxation mechanisms and the use of labor, capital, and other factors to produce goods and services for investment, consumption, and trade. The specification of the production behavior enables health-related labor changes, across all sectors, to be captured and valued at a dynamic wage level which adjusts endogenously according to economic growth. The social accounting matrix (SAM) is the main database used to calibrate the CGE model and was based on the 2004 Ghana SAM which relies on the most recent core Supply–Use Table data.^25^ A dynamic simulation was used to target 2005–2014 macroeconomic indicators and establish 2014 as the new base year, and the counterfactual (baseline) 20 year growth path, for 2015–2034, was constructed to target 2006–2010 historical Ghana growth rates for nominal gross domestic product (GDP) (25.4% per annum [p.a.]) and real GDP (6.6% p.a.).^22^ The counterfactual growth rates and the implied GDP deflator (which grows at 17.4% p.a.) also match recent experience.^26,27^ The relevance of the CGE method, with its recursive dynamic, multi-sector, household-level modeling approach, and its potential to capture productive labor supply impacts to estimate disease burdens have been highlighted, previously, by the WHO.^28^ The base model has been significantly developed to form a fully integrated macroeconomic, epidemiological, demographic model framework, the technical aspects of which are fully documented and reported elsewhere.^17^ Those elements of the model which are of primary relevance to the analyses in this article are briefly outlined in the following subsections.

### Measurement and context.

Our integrated model framework is generalizable, but country contexts vary in terms of their potential for the achievement of elimination and the barriers to elimination. Currently, malaria elimination strategies are being pursued in several African nations, most of which have geographical barriers to reintroduction and/or climatic conditions favoring elimination strategies; for example, Botswana, Comoros, Namibia, South Africa, and Swaziland.^29^ Our study focuses on the less favorable elimination context of Ghana which captures seasonality and takes a more nuanced approach to malaria transmission dynamics. However, the selection of Ghana as the country context further highlights the importance of our subregional analysis.

### Sub-country analysis.

Measurement of the effectiveness of malaria control has most recently focused on a statistically significant association between expanded coverage of prevention measures and reductions in *Pf* malaria prevalence in the 2- to 10-year-old reference age-group (*PfPR*_2–10_) across Africa.^10^ However, the strength of the link is disputed.^9^ Furthermore, when modeling malaria elimination, capturing low/high areas of transmission, at the sub-country level, is important. Thus, our integrated model uses 19 regional malaria transmission models including the Greater Accra Metropolitan Area and 18 other regions which are disaggregated by ecological zone (coastal, forest, and savannah); rural and urban locations; and low–, medium–, and high–malaria transmission locations. This regional division applies not only to the integrated epidemiological malaria transmission models and demographic population models but also to the representative households in the multi-sector CGE model which were derived from disaggregation of households in the underlying SAM data set based on consumption shares from the 2005/2006 Ghana Living Standards Survey - Report 5 (GLSS5) household survey.^30^

### Integrating demographics and epidemiology.

The demographic sub-module captures annual population progression for 1-year age-groups at the regional level. It is based on UN population projections, which were regionalized, to match our 19 household categories, using population shares from the 2005/2006 GLSS5 household survey.^30^ Migration between regions is also specified in our integrated demographic model and is based on census data.

**Figure 2.**
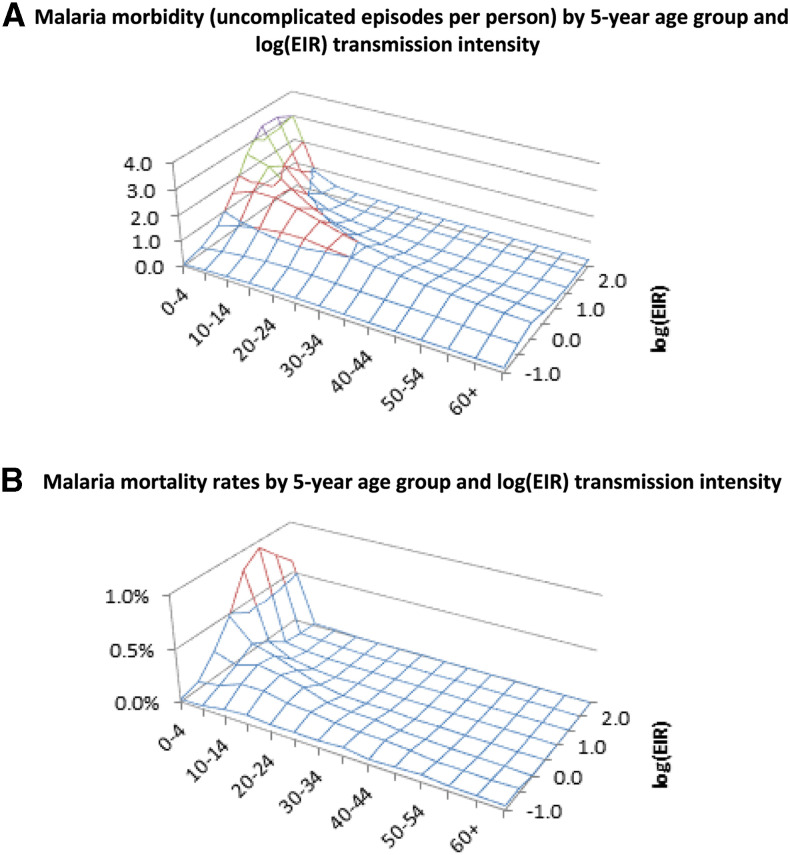
(**A**) Malaria morbidity (uncomplicated episodes per person) by 5-year age-group and log entomological inoculation rate (EIR) transmission intensity. (**B**) Malaria mortality rates by 5-year age-group and log (EIR) transmission intensity. This figure appears in color at www.ajtmh.org.

Each of our regional epidemiological sub-models are a re-specification of a continuous-time epidemiological model as a biweekly discrete time model, which allows for integration with our annually recursive regional demographic and macroeconomic sub-models. The regional models, which, in the MacDonald Ross tradition, use two compartments of human and vector populations,^31^ were extended to account for human superinfections^32^ and calibrated using region-specific human prevalence rates and entomological inoculation rates (EIRs) (the key biomarker for malaria transmission intensities), derived from the Malaria Atlas Project database.^33^ Remaining parameter values were derived from the literature.^34–36^ Clinical outcomes, within the integrated model, were calculated, endogenously, based on lookup tables derived from the STI model.^23^ The STI model includes an age-specific link between clinical outcomes and EIRs, and this allows the model to also capture the relationship between transmission intensity and immunologic stimulation or “semi-immunity”. These nonlinear features of the relation between transmission intensity and clinical outcomes, embodied in the underlying STI model, are also captured in our model framework via a set of piecewise linear specifications, based on the aforementioned simulated lookup tables from the STI model. Surface figures of the lookup tables for morbidity and excess mortality associated with *P. falciparum* infection are provided in Figure 2A and B, respectively, for 12-month all-year transmission. The surface plots show the non-linear relationship between transmission intensity, measured by log-transformed EIRs (log EIRs), and clinical health outcomes (morbidity and mortality) resulting from semi-immunity. The plot indicates that as transmission intensity falls, morbidity and mortality fall among children, but a swell emerges for middle and older age-groups. More detail on the technical specification of the model framework and its sub-components and tables of parameter estimates are available in the full documentation article.^17^

### Capturing private behavior.

A key issue, which has been outlined previously as important for the assessment of growth and scaling up of interventions, is the need for separation, and separate modeling, of private behavior and exogenous policy shocks. Incentive-based private behavior is important for policy analysis and includes rural–urban migration between high- and low-prevalence regions, and adoption of malaria interventions, such as artemisinin-based combination therapies and ITNs. Household decisions closely correlate with the expected income levels. Although they are critical to the impact of economic development on malaria elimination, the combined effects of these income-based incentives have not been studied previously.

Prior evidence on individual mechanisms, by themselves, suggests that economic incentives could play a role in malaria control and elimination.^37,38^ Our integrated framework addresses the combined picture by allowing economic incentives from the macroeconomic CGE sub-model to affect both regional demographic sub-models (through migration incentives), regional *Pf* epidemiological sub-models (through private intervention demands for ITNs), and regional labor market morbidity impacts (through private demand for artemisinin-based combination therapies). These impacts, subsequently, combine to produce predictions of labor force impacts and pecuniary intervention costs which feed back into the macroeconomic model. Because these endogenous feedback effects are specified, separately, for each of our 19 regional household types, the model allows for intervention strategies to have region-specific impacts and enables economy-wide disease burden assessments to account for regional variation in transmission intensities and clinical outcomes as presented earlier. Further technical details on the implementation of these methods are presented elsewhere.^17^

### Estimating disease burden.

To measure the potential future twin health and economic burdens of malaria and assess the potential future benefits of control and elimination efforts, we simulated health and macroeconomic burdens which could be averted over the two coming decades if elimination was achieved in the base year. The method to accomplish this involved running policy scenarios, under the assumption that malaria prevalence/transmission is zero over our 20-year time horizon (2015–2034), and comparing the impacts to our baseline counterfactual growth scenario, based on counterfactual malaria prevalence/transmission patterns. Further details of this methodology are provided elsewhere.^39^

### Modeling scenario: economic growth.

The integrated nature of our model, where changes in household income may provoke migration and changes in private demand for interventions, is the critical feature which allows us to analyze the impact of changes in economic growth on epidemiological, demographic, and clinical health outcomes, and thereby to study whether, and to what extent, economic growth can assist in controlling, and ultimately eliminating, malaria. Specifically, we simulate variations in economic growth rates, ranging between ±2 percentage points per annum and centered around the 6.6% baseline growth rate.

### Modeling scenario: scaling up ITNs.

In the same way as described earlier, our integrated model framework, with its separation, and separate modeling, of private behavior and exogenous policy shocks, allows us to analyze the impact of changes in public scaling up of preventive interventions on epidemiological, demographic, and clinical health outcomes, and thereby to study whether, and to what extent, such scaling up can assist in controlling, and ultimately eliminating, malaria. Specifically, we simulate the scaling up of ITN coverage rates by up to 25 percentage points relative to counterfactual levels.

### Sensitivity analysis.

Computable general equilibrium models are deterministic and are calibrated with economic data which are not statistically estimated. As a consequence, sensitivity analyses are used, extensively, to test key assumptions. In our case, a key assumption relates to the uptake of ITNs, which are used to determine effective coverage rates in the model. Documentation of uptake and coverage rates is provided in the documentation paper^22^ and tabulated in the Supplemental Materials. To test the sensitivity of our model to changes in uptake rates, we ran simulations for low uptake (by halving uptake rates for all regional households) and high uptake (by doubling uptake rates for all regional households).

## RESULTS

**Figure 3.**
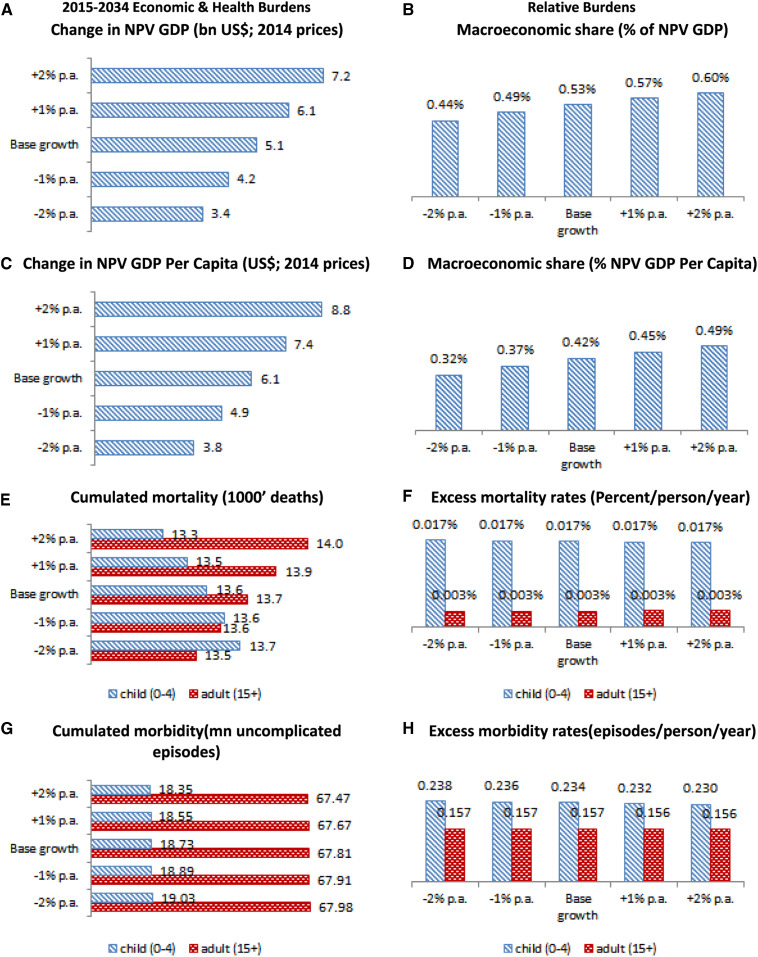
2015–2034 Malaria disease burdens in Ghana: The impact of economic development. (**A**) Change in NPV GDP (billion US$; 2014 prices). (**B**) Macroeconomic share (% of NPV GDP). (**C**) Change in NPV GDP per capita (US$; 2014 prices). (**D**) Macroeconomic share (% NPV GDP per capita). (**E**) Cumulated mortality (1,000 deaths). (**F**) Excess mortality rates (percent/person/year). (**G**) Cumulated morbidity (million uncomplicated episodes). (**H**) Excess morbidity rates (episodes/person/year). Change in NPV GDP = change in net present value of GDP (accounting for the future value of money); % of NPV GDP = percentage of net present value of GDP (accounting for the future value of money); p.a. = per annum. This figure appears in color at www.ajtmh.org.

To express the cumulative macroeconomic burden of malaria as a time value of future GDP, we apply a 5.0% real discount rate to future 2015–2034 macroeconomic burden estimates and sum them. This valuation is labeled as net present value (NPV). The baseline changes in NPV of GDP (or “NPV GDP”), due to the elimination of the malaria disease burden, are presented in Figure 3A in billion US$, whereas the relative impacts, as a percentage of baseline total NPV GDP, are presented in Figure 3B. These results show that if Ghana continues along its current economic growth path over the coming 20 years, the cumulative macroeconomic burden of malaria will amount to US$5.1 billion (in 2014 prices) or around 0.5% of the NPV of future GDP. Without additional public action, such as increased domestic use of ITNs, the cumulative health burden will include an estimated 43,400 deaths and 123.4 million uncomplicated malaria episodes.^39^

Because our integrated CGE model captures true productivity losses and spillover effects, valued at dynamic wage rates, these results cannot be readily compared with alternative valuation methodologies, and CGE studies of malaria are rare. However, for comparison, there is one CGE modeling study, which excludes health modeling, but which has, nonetheless, been used to estimate hypothetical malaria intervention effects in Ghana.^40^ The latter study shows that a hypothetical treatment of children with 100% coverage, 50% efficacy against clinical malaria, and 20% efficacy against malaria mortality might yield economic gains of US$6.93 billion or 0.5% in 30 years. The results after 20 years estimated a GDP gain of approximately 0.3%, which is approximately half our estimates of the macroeconomic disease burden. More detailed analyses on disease burdens, using our integrated methodology, have, as noted previously, been conducted and are available elsewhere.^39^

### Linkages between malaria and economic growth.

Economic growth is strongly correlated with the health burden from malaria, which is expected given the importance of labor to the economy. However, contrary to popular belief, the correlation is positive: reduced future economic growth lowers the cumulative economic burden from malaria by as much as one-third from US$5.1 billion to US$3.4 billion (Figure 3A), whereas increased economic growth could significantly increase the economic burden to US$7.2 billion. This is attributable to wage effects: one of the strengths of CGE modeling is its ability to capture dynamic changes in wages, over time, in response to changes in the economy, and in our integrated model framework, this manifests itself in our ability to undertake model-consistent valuation of workforce reductions attributable to future changes in malaria disease burdens. Wage levels generally increase with economic growth because of higher marginal returns to labor, and the loss of labor, attributable to malaria, therefore carry a larger value in a growing economy. Economic growth also increases prevention-related demand for ITNs, which, in turn, affects morbidity and mortality. However, the health-related changes in workforce participation rates, which stem from increased demand for and use of ITNs, are not sufficiently large to counter the dominating wage impact on the macroeconomic burden. Results from our simulations, presented in Figure 3, show mortality impacts as the number of deaths (Figure 3E), morbidity impacts as the number of uncomplicated episodes (Figure 3G), and morbidity and mortality rates per person per year (Figure 3F and H). These results indicate that increased economic growth only marginally affects clinical outcomes, including child mortality (≈−2%) and adult mortality (≈+2%). The counter-intuitive impact on adult mortality is attributable to interaction effects of economic growth. As mentioned earlier, higher economic growth increases wages and provokes increased uptake of ITNs, and this has a positive health impact on younger population members by reducing exposure before adulthood. However, lower exposure among younger population members prevents the acquisition of semi-immunity, something which is only acquired after exposure to the parasite. Thus, decreased transmission that fails to achieve elimination will produce a demographic shift which may result in increased mortality among adults whose lower exposure to malaria as children has prevented them from acquiring semi-immunity and left them more susceptible to the clinical effects of malaria in adulthood, a phenomenon which has been previously proposed but not quantified.^41^

Because our model estimates that increased economic growth increases the economic NPV GDP disease burden more than it increases the population (due to higher marginal productivity of diseased workers), it is not surprising that measurements of economic disease burden, expressed as NPV GDP per capita and shown in US$ per capita, in Figure 3C, and in relative terms, in Figure 3D, also increase. Our baseline estimate of the NPV GDP per capita measure of disease burden is US$6.1 (0.4% of GDP per capita), but increasing/decreasing growth by 2 percentage points yields estimates of US$8.8 (0.5%)/US$3.8 (0.3%), respectively, indicating that the counter-intuitive effects of economic growth, on the economic burden of malaria, will be felt at both the national and individual levels. The ripple effects of economic development are, therefore, likely to represent a set of policy challenges for both economic and health authorities in hyperendemic countries such as Ghana.

**Figure 4.**
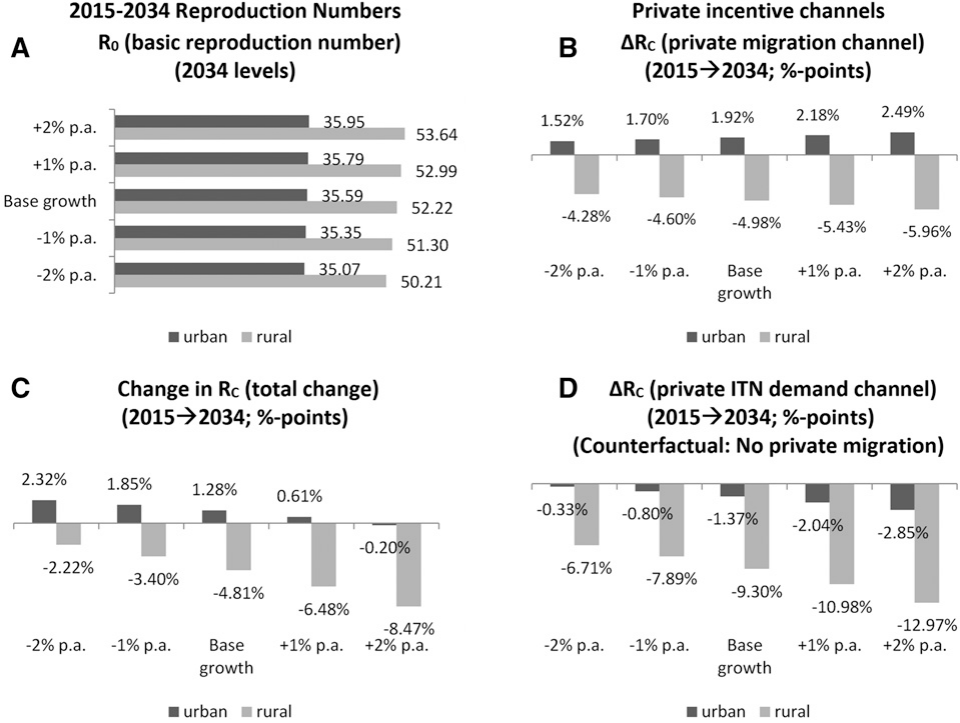
2015–2034 Malaria reproduction numbers in Ghana: The impact of economic development and private incentive channels. (**A**) *R*_0_ (basic reproduction number) (2034 levels). (**B**) ∆*R*_C_ (private migration channel) (2015–2034; percentage points). (**C**) Change in *R*_C_ (total change) (2015–2034; percentage points). (**D**) ∆*R*_C_ (private ITN demand channel) (2015–2034; percentage points) (counterfactual: no private migration). *R*_0_ = reproductive number; ∆*R*_C_ = change in controlled reproductive number; p.a. = per annum.

**Figure 5.**
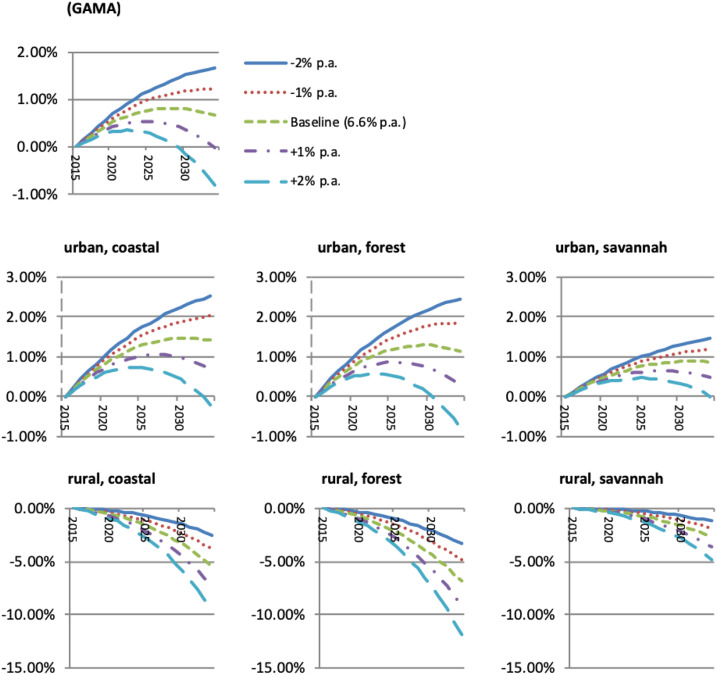
Economic development impact on controlled regional reproduction numbers (change in *R*_C_). GAMA = Greater Accra Metropolitan Area; *R*_C_ = controlled reproductive number; p.a. = per annum. This figure appears in color at www.ajtmh.org.

Overall, integrated modeling suggests that economic development is a weak tool for malaria control. Future reproduction numbers *R*_0_ will be relatively unaffected (Figure 4A), and although controlled reproduction numbers *R*_C_ will decline moderately in rural areas by up to 8.5% because of increased urbanization in the high growth scenario, they will be relatively unaffected elsewhere (Figure 4C). Without additional intervention, regional *R*_C_ values would, therefore, remain high over the next 20 years for all regions of Ghana (*R*_C_ ≈ 35–55 on average; *R*_C_ > 12 for all subregions; see Figure 5), regardless of whether economic growth improves or deteriorates. Economic growth also has some impact on malaria prevalence: our simulated countrywide prevalence estimate of 56.9% in 2015 decreases to 54.1% by 2034 in the baseline scenario (not shown). However, our lower (−2%) growth scenario results in a smaller reduction to 55.8% by 2034, whereas our upper (+2%) growth scenario reduces prevalence to 51.7% by 2034. Our results suggest, therefore, that economic development cannot be relied on to achieve external malaria elimination. To impact positively on malaria elimination, economic growth must produce sufficiently strong incentives on individual behavior to invoke a willingness to purchase ITNs (Figure 4B) and to migrate from high- to low-transmission areas (Figure 4D). Although growing income levels will raise private ITN demand and outward immigration will further increase effective ITN coverage rates for the remaining rural population, the direction of migration patterns will also put pressure on urban areas.

### Impact of scaling up ITNs.

**Figure 6.**
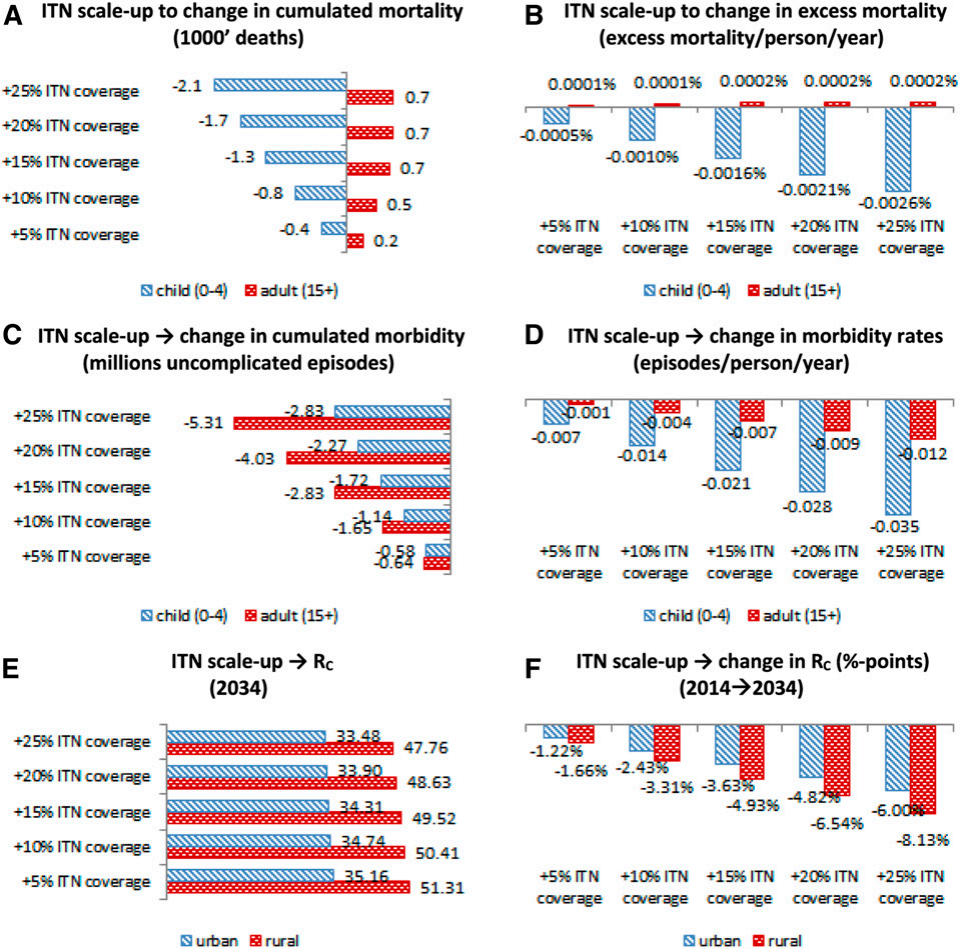
2015–2034 Public action: ITN scaling up (morbidity, mortality, and control). (**A**) Insecticide-treated net scaleup to change in cumulated mortality (1,000 deaths). (**B**) Insecticide-treated net scaleup to change in excess mortality (excess mortality/person/year). (**C**) Insecticide-treated net scaleup to change in cumulated morbidity (million uncomplicated episodes). (**D**) Insecticide-treated net scaleup to change in morbidity rates (episodes/person/year). (**E**) Insecticide-treated net scaleup to *R*_C_ (2034). (**F**) Insecticide-treated net scaleup to change in *R*_C_ (percentage points) (2014–2034). ITN = insecticide-treated net; *R*_0_ = reproductive number; *R*_C_ = controlled reproductive number. This figure appears in color at www.ajtmh.org.

Because economic growth may have a limited, if not negative, effect on the elimination of malaria, what about the role of external public preventive interventions such as public procurement of ITNs? Although some research has suggested that malaria control and elimination in Africa can be achieved by mass procurement and distribution of ITNs,^10^ the scale of predicted reductions in malaria transmission intensities at the regional level are not supported by our structural modeling for Ghana. Our results suggest that a future public campaign to increase regional ITN coverage rates by 25 percentage points will lower malaria reproduction numbers by at most 9% (Figure 6F). This reduction in reproduction numbers is accompanied by a cumulative reduction in morbidity of 2.83 million episodes (0.035%), among children, and 5.31 million episodes (0.012%), in total, over our 20-year simulation period (Figure 6C and D). In terms of prevalence, our simulations suggest that scaling up ITN coverage by 25% will reduce malaria prevalence, from 54.1% to 45.9%, in 2034 (not shown), but will not, by itself, bring elimination within reach.

**Figure 7.**
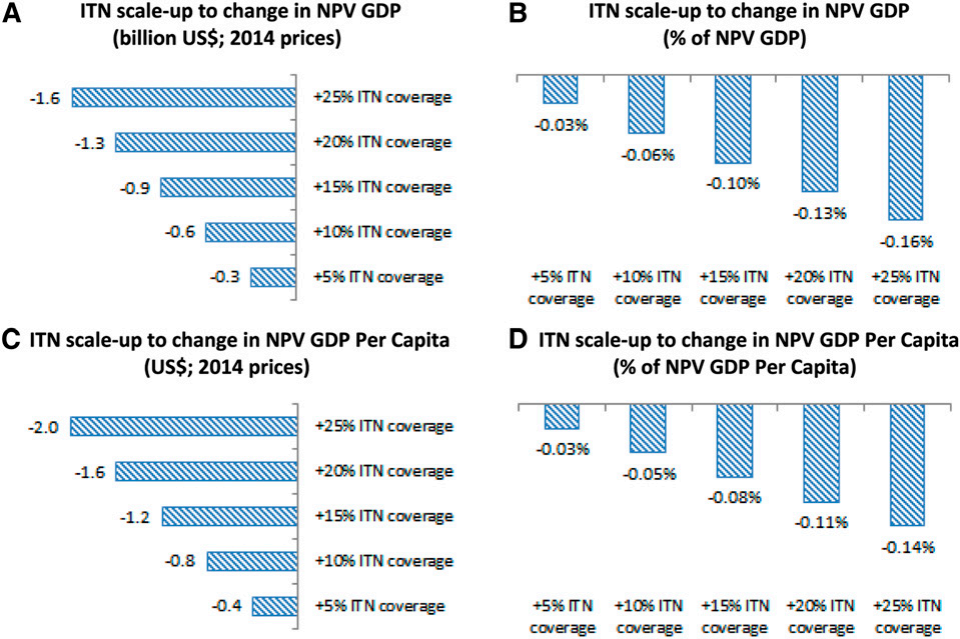
2015–2034 Public action: ITN scaling up (economic). (**A**) Insecticide-treated net scaleup to change in NPV GDP (billion US$; 2014 prices). (**B**) Insecticide-treated net scaleup to change in NPV GDP (% of NPV GDP). (**C**) Insecticide-treated net scaleup to change in NPV GDP per capita (US$; 2014 prices). (**D**) Insecticide-treated net scaleup to change in NPV GDP per capita (% of NPV GDP per capita). ITN = insecticide-treated net; NPV GDP = net present value of GDP (accounting for the future value of money). This figure appears in color at www.ajtmh.org.

However, on the economic side, our structural analysis further suggests that increasing coverage of ITNs will be expensive and, unless the large (and recurring) costs of such campaigns are funded by external partners, the increased economic burden of scaleup could amount to US$1.6 billion (in 2014 prices) or around 0.2% of the future NPV GDP (Figure 7A and B). Furthermore, if public ITN distribution campaigns were used to increase ITN coverage, our analysis predicts (partially) adverse effects on child and adult mortality, similar to growth-driven private ITN demand (Figure 6B). Whereas excess mortality rates may decline by 0.0026 percentage points, for children younger than 5 years, by 2034, the reduced semi-immunity, carried into adulthood, could increase adult excess mortality rates by 0.0002 percentage points (Figure 6B). The expense of scaling up interventions is also reflected in NPV GDP per capita estimates (Figure 7C and D). Although scaling up of ITN coverage rates, by 25 percentage points, has a positive population-wide impact on mortality (reducing child deaths by 2,078 and increasing adult deaths by 726), the overall effect on NPV GDP per capita is a reduction of US$2.0, or 0.1%, and our results suggest that these impacts are approximately linear such that for every 5% increase in ITN coverage, GDP per capita will decline by US$0.4.

### Sensitivity analyses.

**Figure 8.**
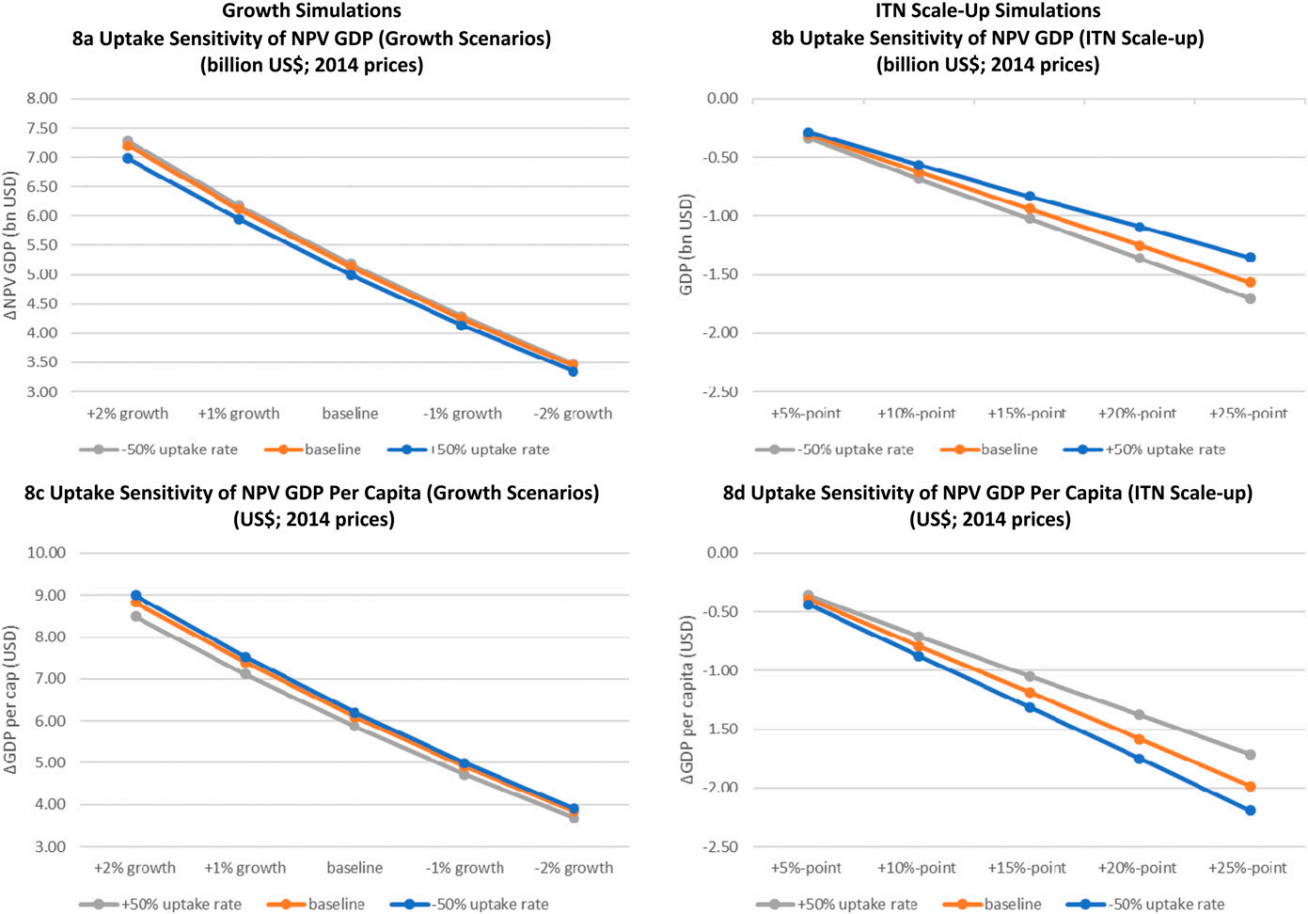
GDP per capita. (**A**) Uptake sensitivity of NPV GDP (growth scenarios) (billion US$; 2014 prices). (**B**) Uptake sensitivity of NPV GDP (ITN scaleup) (billion US$; 2014 prices). (**C**) Uptake sensitivity of NPV GDP per capita (growth scenarios) (US$; 2014 prices). (**D**) Uptake sensitivity of NPV GDP per capita (ITN scaleup) (US$; 2014 prices). ITN = insecticide-treated net; NPV GDP = net present value of GDP (accounting for the future value of money). This figure appears in color at www.ajtmh.org.

Sensitivity analyses of our growth and ITN scaling up scenarios, for low (half) and high (double) ITN uptake rates, are shown in Figure 8A–D. The results indicate that doubling uptake rates reduces NPV GDP estimates of disease burdens by approximately 3% for all growth scenarios and halving uptake rates increases NPV GDP estimates of disease burden by approximately 1%.

Applying a similar set of sensitivity analyses to our ITN scaling up scenarios reveals, not surprisingly, that as the provision of ITNs increases, the effect of varying uptake rates also increases. Although halving/doubling uptake rates causes −3%/+1% variations in NPV GDP burden estimates at baseline coverage rates, it causes −14%/+9% impacts on GDP burden estimates for the scenario where ITN coverage is increased by 25 percentage points. Relative variations in NPV GDP per capita are similar to the changes in NPV GDP, indicating that sensitivity to variations in uptake rates is similar at the national and individual levels. The economic disease burden impacts of the aforementioned sensitivity scenarios are tabulated in the Supplemental Materials.

## CONCLUSION

Our structural modeling for Ghana, which accounts for both epidemiological and demographic interactions, suggests that neither economic development nor public ITN scaling up efforts alone will be sufficient to eliminate malaria. Our analysis further suggests that private incentive mechanisms, in the form of internal migration and private ITN demand, which affect *R*_C_ rather than *R*_0_, will lower reproduction numbers by less than 6% over the coming 20 years, and public ITN distribution campaigns will have a similar limited capacity to interrupt malaria transmission. Indeed, in the absence of malaria elimination, near-term ITN scaleup efforts may shift the demographic burden to older age-groups and actually increase malaria-attributable deaths in adult populations. This calls into question whether universal ITN coverage alone can be relied on to eliminate malaria in a hyperendemic country such as Ghana and echoes the concerns raised by recent cluster-randomized trials of ITNs and indoor residual spraying (IRS) in low-transmission settings which also question the potential for ITNs, IRS, or a combination of those interventions to achieve elimination.^8^

As countries work toward the Sustainable Development Goals, sub-Saharan Africa is at the crossroads where policymakers increasingly strive for malaria elimination but where present tools to combat malaria may not be sufficient by themselves to achieve the objective. Where does this leave us?

On the one hand, external factors which cannot be captured in our model may influence the magnitude of effects that have been presented in this study. For example, urbanization may result in improvements in drainage and housing which affect mosquito-breeding grounds and reduce human–vector contact, and because these external factors may affect all ages and be sustained, they may be less subject to the semi-immunity effects which accompany scaling up of ITNs and increase adult mortality.^15^ However, obtaining data to parameterize the extent to which multifaceted interventions could achieve malaria elimination in Ghana goes beyond the scope of this study. In the case of malaria in the United States, for example, multiple direct and indirect factors have been cited as contributing to successful eradication including a long-term malaria education campaign, growth of health departments with focused malaria control, road and transportation improvements, development of improved canal systems, and large-scale use of larvicides followed by the Extended Malaria Control Program, Malaria Eradication Program, and malaria surveillance.^42^ In addition to these complex direct and indirect factors, external effects such as drug resistance could hamper treatment, multiplying the health and macroeconomic effects of mortality, and climate change has also been cited as a potential confounder of the positive effects of socioeconomic development on malaria elimination.^43^ However, although some research indicates that climate change may provoke a net increase in population risk of malaria, they also highlight that there are large uncertainties and variations between models.^44^ By contrast, other spatial studies have indicated that central climate change scenarios have a negligible effect on malaria prevalence (*PfPR*_2–10_) by 2050.^45^ Given the complex nature of our integrated model, we have not attempted to capture these effects.

In conclusion, our modeling does not support the hypothesis that malaria elimination in a hyperendemic environment, such as Ghana, can be achieved using current tools accompanied by economic growth. In short, the efficacy of our present-day malaria control tools, in malaria-endemic areas, depends on the degree of semi-immunity among the resident populations in these areas. Research suggests that improved vector control technologies are possible and, in their current form, could provide affordable and more effective control in areas with high population density.^46^ Future investment to improve technology and/or delivery of these tools may advance progress toward elimination. Research to inform and refine guidelines for vector control are ongoing and may enable the maximization of control and progress toward elimination within resource-limited environments.^47^ However, regardless of how tools and implementation methods may improve and change in coming years, previous experience dictates that current levels of malaria control must be maintained and resurgence avoided, until malaria elimination is accomplished.

Perhaps, the most viable route for accomplishing malaria elimination is the development of new tools and technologies that significantly reduce transmission and/or infection. For example, the RTS,S pre-erythrocytic vaccine against *Pf* malaria infections has been shown to provide 25–50% protection for infants over extended periods of time in clinical trials, and pilot implementation of this vaccine is ongoing in Ghana and two other pilot countries as part of routine immunization services.^48,49^ Although it has been stated that vaccines which are currently under development and targeting parasitic diseases “cannot be compared to the well-known highly efficacious vaccines of the childhood diseases caused by bacteria and viruses,”^50^ partially efficacious vaccines may show a significant alleviation of the disease burden and, thus, illustrate the potential for future technological developments to facilitate rapid progress toward elimination beyond that which can be achieved by other existing control measures.

Given the ineffectiveness of economic development and the hard trade-offs presented by current vector control interventions, future research and development of more effective vector and disease control technologies and strategies for application/dissemination should be pursued with intensity. However, it is concerning that international donor support, in the fight against malaria, has shown signs of stagnation, and recent evidence suggests that although US$588 million (85% of the estimated annual need) was spent on research and development in 2016, research and development funding for malaria vaccines and drugs declined in 2016 compared with that in 2015.^1^ In addition, the Sustainable Development Goals, though important, are more diffuse than the Millennium Development Goals and risk diluting financial support to the health sector. Thus, reinforcement of current malaria control measures and increased investment to develop efficacious tools for malaria elimination are more urgent than ever.^49^ Our research suggests that current interventions are unlikely to achieve malaria elimination. This conclusion is supported by the recent Lancet Commission which suggests that persistence of malaria in 10 countries (including Ghana) is a barrier to eradication and cannot be achieved under a business-as-usual scenario or with current tools alone. However, the report indicates that eradication is achievable by 2050 by “improving management and operations and making better use of existing technologies, rolling out new technologies, and spending more money” but, in contrast, failure to pursue this goal would be “indefensible.”^45^ Unrelenting support for malaria control alongside increased investment in elimination-targeted research and development is therefore essential to maintain current gains against malaria to avoid a resurgence in malaria transmission and malaria-attributable mortality and, ultimately, to build on current progress to accomplish elimination.

## Supplemental materials

Supplemental materials
