# Supplementary material for: Will More of the Same Achieve Malaria Elimination? Results from an Integrated Macroeconomic Epidemiological Demographic Model
Source: Am J Trop Med Hyg. 2020 Sep 21;103(5):1871–82. doi: 10.4269/ajtmh.19-0472 (PMC7646798; doi:10.4269/ajtmh.19-0472)
Supplement: Supplementary file 1 [file tpmd190472.SD1.doc]

**The following are supplemental files and will be published online only**

**Supplemental Material**

S1: ITN Uptake rates

The household-specific estimates of coverage and ITN uptake rates were computed from a range of data sources. Household-specific coverage and uptake rates were derived from the 2014 Ghana Demographic and Health Survey (GHS 2015), while household-specific splits between private and public coverage rates were based on ‘public sector’ and ‘public campaign’ coverage estimates from the fourth round 2012 MICS4 Multiple Indicator Cluster Survey (GSS 2012). A single average cost estimate for ITNs, computed as the weighted average of 'Public', 'Private' and 'Other' median costs from the 2012 MICS4 Survey (ibid.), was attributed to each of our 19 household types since no household-specific information was available.

Table S1: Variable initialization and parameter values for ITN Uptake rates

|  | Coverage rates  (percent) | |  | Uptake rates  (percent) |  |
| --- | --- | --- | --- | --- | --- |
| Household | private | public/free |  |  |  |
| Low prevalence GAMA | 16.3% | 17.0% |  | 49% |  |
| Low prevalence Urban Coastal | 14.2% | 22.9% |  | 75% |  |
| Med prevalence Urban Coastal | 14.2% | 22.9% |  | 75% |  |
| HIgh prevalence Urban Coastal | 14.2% | 22.9% |  | 75% |  |
| Low prevalence Urban Forest | 18.5% | 24.1% |  | 70% |  |
| Med prevalence Urban Forest | 18.5% | 24.1% |  | 70% |  |
| HIgh prevalence Urban Forest | 18.5% | 24.1% |  | 70% |  |
| Low prevalence Urban Savannah | 10.9% | 20.1% |  | 78% |  |
| Med prevalence Urban Savannah | 10.9% | 20.1% |  | 78% |  |
| HIgh prevalence Urban Savannah | 10.9% | 20.1% |  | 78% |  |
| Low prevalence Rural Coastal | 13.2% | 30.0% |  | 128% |  |
| Med prevalence Rural Coastal | 13.2% | 30.0% |  | 128% |  |
| HIgh prevalence Rural Coastal | 13.2% | 30.0% |  | 128% |  |
| Low prevalence Rural Forest | 18.1% | 31.4% |  | 120% |  |
| Med prevalence Rural Forest | 18.1% | 31.4% |  | 120% |  |
| HIgh prevalence Rural Forest | 18.1% | 31.4% |  | 120% |  |
| Low prevalence Rural Savannah | 9.7% | 26.2% |  | 134% |  |
| Med prevalence Rural Savannah | 9.7% | 26.2% |  | 134% |  |
| HIgh prevalence Rural Savannah | 9.7% | 26.2% |  | 134% |  |

N.B. Coverage rates express the percentage of households with access to ITNs. Where uptake rates are larger than 100% this indicates that there is, on average, more than one person sharing each net.

**References:**

GHS (Ghana Health Service). 2015. Ghana Demographic and Health Survey. *Ghana Statistical Service and Ghana Health Service*, Accra.

GSS (Ghana Statistical Services). 2012. Multiple Indicator Cluster Survey, round 4 (MICS4). *Ghana Statistical Services*. Accra.

**S2: Sensitivity of Uptake Results (GDP)**

Growth Scenarios

| **∆NPV GDP impacts (bn USD):** | | |  |  |  |
| --- | --- | --- | --- | --- | --- |
|  | +2% growth | +1% growth | baseline | -1% growth | -2% growth |
| +50% uptake rate | 6.99 | 5.95 | 5.00 | 4.13 | 3.35 |
| baseline | 7.20 | 6.12 | 5.14 | 4.25 | 3.45 |
| -50% uptake rate | 7.29 | 6.18 | 5.19 | 4.29 | 3.48 |
| **∆NPV GDP per capita impacts (USD):** | | | |  |  |
|  | +2% growth | +1% growth | baseline | -1% growth | -2% growth |
| +50% uptake rate | 8.49 | 7.12 | 5.87 | 4.72 | 3.68 |
| baseline | 8.83 | 7.40 | 6.10 | 4.91 | 3.84 |
| -50% uptake rate | 9.00 | 7.53 | 6.20 | 4.99 | 3.91 |

Growth Scenarios

| **∆NPV GDP impacts (bn USD):** | | |  |  |  |
| --- | --- | --- | --- | --- | --- |
|  | +5%-point | +10%-point | +15%-point | +20%-point | +25%-point |
| +50% uptake rate | -0.28 | -0.56 | -0.83 | -1.09 | -1.35 |
| baseline | -0.31 | -0.63 | -0.94 | -1.25 | -1.56 |
| -50% uptake rate | -0.34 | -0.68 | -1.02 | -1.36 | -1.70 |
| **∆NPV GDP per capita impacts (USD):** | | | |  |  |
|  | +5%-point | +10%-point | +15%-point | +20%-point | +25%-point |
| +50% uptake rate | -0.36 | -0.71 | -1.05 | -1.38 | -1.71 |
| baseline | -0.39 | -0.79 | -1.19 | -1.58 | -1.98 |
| -50% uptake rate | -0.44 | -0.87 | -1.31 | -1.75 | -2.19 |
